# Amniotic Membrane-Derived Factors in Immunomodulation and Regenerative Medicine: Current Evidence and Emerging Perspectives in Biomaterials and 3D Bioprinting

**DOI:** 10.3390/jfb17070321

**Published:** 2026-07-04

**Authors:** Hristina Obradović, Ivana Gazikalović, Ivana Okić Đorđević, Sanja Momčilović, Dragana Aleksandrović, Nikola Jeftić, Aleksandra Jauković

**Affiliations:** 1 Group for Hematology and Stem Cells, Institute for Medical Research, National Institute of Republic of Serbia, University of Belgrade, 11000 Belgrade, Serbia; ivana.okic@imi.bg.ac.rs (I.O.Đ.); dragana.aleksandrovic@imi.bg.ac.rs (D.A.); aleksandra@imi.bg.ac.rs (A.J.); 2 Innovation Center of the Faculty of Technology and Metallurgy, University of Belgrade, 11000 Belgrade, Serbia; igazikalovic@tmf.bg.ac.rs; 3 Group for Neuroendocrinology, Institute for Medical Research, National Institute of Republic of Serbia, University of Belgrade, 11000 Belgrade, Serbia; sanja.momcilovic@imi.bg.ac.rs; 4 Institute for Oncology and Radiology of Serbia, 11000 Belgrade, Serbia; jefta20@gmail.com

**Keywords:** amniotic membrane, 3D bioprinting, biomaterials, regenerative medicine, tissue engineering, immunomodulation

## Abstract

Human placenta-derived amniotic membrane (hAM) and its derivatives have attracted growing interest as bioactive materials for tissue engineering, regenerative medicine, and biomaterial design. This review summarizes the anatomical and cellular characteristics of hAM and examines the interplay between its regenerative and immunomodulatory properties. Key hAM-derived biomolecules are discussed, with an emphasis on their roles in immune regulation, angiogenesis, extracellular matrix remodeling, and tissue repair across diverse regenerative contexts. Current applications of hAM-based materials in tissue engineering and regenerative biomaterials are reviewed, including emerging studies involving soft tissue applications. In addition, recent efforts to integrate hAM derivatives into 3D bioprinting approaches are examined, including their use as bioink components or biofunctional additives to hydrogel-based systems. Although studies involving hAM-based bioprinted constructs remain limited, the available findings suggest promising regenerative, proangiogenic, and bioactive effects. Challenges related to material processing, printability, standardization, and reproducibility are also discussed. Overall, this review highlights the biomaterial potential of hAM derivatives and their emerging relevance to biofabrication strategies aimed at developing biologically instructive constructs for regenerative medicine.

## 1. Introduction

The field of tissue engineering, as the main tool in regenerative medicine, is a relatively young yet extensively investigated field of biomedicine. With the main goal of stimulating tissue regeneration without side effects, in recent years, researchers have focused on developing various solutions by using natural compounds. These include but are not limited to plant and animal polymers. In more than a decade, perinatal tissues (placenta, umbilical cord and amniotic fluid) have been suggested and explored as favorable tools in tissue engineering and beyond thanks to their remarkable features. Placenta and other perinatal tissues are mostly discarded after birth, which makes them available and ethically non-compromised, and, besides that, they are minimally immunogenic, both suitable traits for biomaterial development. Moreover, placental derivatives, such as amniotic membrane (AM), are rich in potent cells, bioactive molecules and an extracellular matrix (ECM) that can stimulate regeneration of many tissues, as demonstrated in various preclinical and clinical research. Furthermore, their immunomodulatory [1], antimicrobial [2], antifibrotic [3] and anticancer [4] effects have been documented in a number of studies, further contributing to their therapeutic potential.

Amniotic membrane and its derivatives have mostly been investigated and successfully applied in skin and cornea regeneration, with many successful applications also documented in regeneration of other tissues. Even with success in this field, tissue engineering aims to provide an effective, fast, available and personalized approach, constantly teaming up with other fields of science and introducing advanced methodologies. Additive technologies, such as 3D bioprinting, have been more extensively used and investigated in recent years due to the various advantages of produced scaffolds, tissues, grafts or implants over traditional wound healing methods and scientific investigation. Furthermore, thanks to 3D scanning and modeling, which often, if not always, go hand in hand with 3D bioprinting, this methodology provides the possibility of personalized approaches. The application of 3D bioprinted scaffolds offers a wide variety of applications in the revitalization and reconstruction of soft and hard tissues as part of tissue engineering and regenerative medicine [5,6]. In this review, we aim to address the properties of the human placenta’s AM that make it a suitable source of derivatives that could be used for the development of biomaterials for 3D bioprinting and tissue engineering of soft tissues.

## 2. Immunomodulation as a Cornerstone of Regenerative Medicine

Regenerative medicine represents a dynamic multidisciplinary field that integrates stem cell biology, tissue engineering, and biomaterials science to develop innovative therapies for repair and regeneration of damaged or diseased tissues. The major cornerstone of regenerative medicine is stem cell-based therapy conceptualized to improve tissue repair machinery via stem cell regulation toward regeneration [7]. The mechanisms underlying stem cell-based therapies include direct cell replacement by stem cell differentiation into specialized cell types, as well as paracrine effects of stem cell-derived growth factors, cytokines and immunomodulatory molecules that shape the local microenvironment towards tissue repair.

Although regenerative properties of stem cells have been evidenced, their clinical applications face many challenges besides regulatory hurdles in terms of efficacy. Namely, the natural process of tissue regeneration involves myriad different cell types interacting simultaneously to repair the damaged tissue. Besides stem cells, other stromal and immune cells play a key role in the tissue repair response [8]. To better recapitulate natural tissue repair processes, the importance of an in-depth understanding of intercellular interactions in tissue regeneration is increasingly recognized. This particularly refers to the mutual crosstalk between the immune system and stem/progenitor cells during tissue healing that inspires novel regenerative approaches integrating the aspect of immune modulation.

The contributions of immune response to tissue regeneration are complex, so there are possible negative and positive outcomes (degree of scarring, fibrosis, and organ function restoration) depending on the microenvironment context determined by tissue type, cellular content, age, and health condition [9]. Although the main roles of effector cells of innate and adaptive immunity in regenerative processes have been defined, the mechanisms of immune cell interactions with resident or recruited stem cells in the specific niche of an injured site are still elusive.

**Figure 1:**
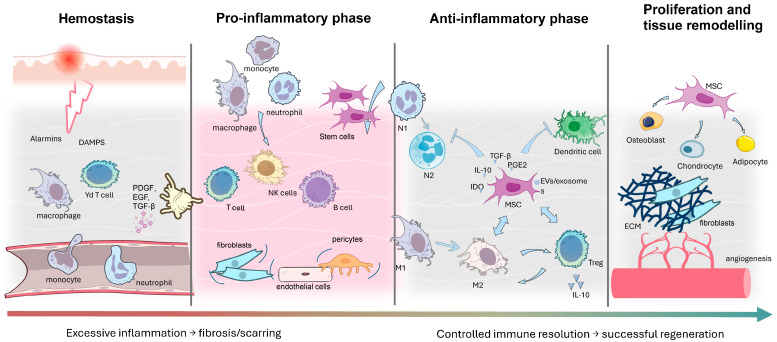
Tissue repair proceeds through an orchestrated sequence of phases: hemostasis, inflammation, anti-inflammatory resolution, and proliferation/remodeling. During hemostasis, platelets respond to tissue damage by forming a clot and releasing PDGF, PF4, EGF, and TGF-β, while resident immune cells detect danger signals (DAMPs) and recruit neutrophils. The pro-inflammatory phase is characterized by infiltration of neutrophils, monocytes, macrophages, and lymphocytes, as well as activation of stromal cells. The anti-inflammatory phase begins as neutrophils and macrophages transition from pro-inflammatory (N1/M1) to anti-inflammatory (N2/M2) phenotypes. Mesenchymal stromal cells (MSCs) critically contribute to this transition by secreting IL-10, TGF-β, IDO, PGE_2_, and extracellular vesicles, promoting regulatory T cell activity and suppressing effector immune responses. During proliferation and remodeling, angiogenesis, extracellular matrix deposition, and MSC differentiation (into osteogenic, adipogenic, or chondrogenic lineages) support tissue regeneration and functional restoration. Dysregulation of these interactions can lead to excessive inflammation and fibrosis, whereas balanced immune–stem cell crosstalk promotes regenerative healing. Illustration made with images provided by Servier Medical Art (https://smart.servier.com), licensed under CC BY 4.0 (https://creativecommons.org/licenses/by/4.0/, accessed on 28 June 2026) and NIAID NIH BioArt Source.

The process of tissue repair, initiated upon injury or damage, includes an orchestrated sequence of events including hemostasis, pro-inflammatory, anti-inflammatory, proliferation and tissue remodeling phases, where the effective completion of each phase warrants optimal tissue healing (Figure 1). During hemostasis, a local response to tissue injury and danger signals (DAMPs and alarmins) is promptly induced by resident immune cells, such as macrophages and γδT cells, which subsequently induce mobilization of other immune cells [10]. Moreover, platelet-secreted factors (platelet-derived growth factor, PDGF, platelet factor 4, epidermal growth factor—EGF, and transforming growth factor-β—TGF-β) recruit neutrophils and monocytes [11]. At this step, a pro-inflammatory phase develops, triggering recruitment, proliferation, and activation of various immune, stromal (fibroblasts, endothelial cells, and pericytes) and stem cells. Namely, the innate immunity cells, including neutrophils, monocytes and macrophages infiltrated at the injured site, stimulate mobilization of adoptive immunity cells (T, B, and natural killer lymphocytes), further influencing immune responses [9]. The resolution of this phase and initiation of the anti-inflammatory phase coincide with the transition of neutrophils and macrophages from pro-inflammatory type-1 (N1 and M1) to anti-inflammatory type-2 phenotype (N2 and M2) [12]. Particularly, anti-inflammatory M2 macrophages contribute to regeneration by influencing regulatory T cells (Tregs), which sequentially sustain the anti-inflammatory M2 phenotype through interleukin (IL)-10 secretion [13]. The following remodeling phase, featuring angiogenesis and ECM component deposition, provides new vessel formation and tissue repair [10]. Besides clearance of cellular debris, the immune cells in this phase facilitate tissue regeneration by affecting proliferation and differentiation of tissue-specific stem and progenitor cells, including mesenchymal stromal/stem cells (MSCs) [8].

MSCs are multipotent adult stem cells featuring the ability to differentiate into multiple cell types of mesodermal origin (osteoblasts, chondrocytes, and adipocytes) [14]. They also possess broad immunomodulatory properties and secrete a range of bioactive factors, including growth factors, chemokines, anti-inflammatory cytokines, immunosuppressive mediators and extracellular vesicles, which support tissue repair and regeneration [15,16,17,18,19]. Through these secretions, MSCs regulate both innate and adaptive immune responses during tissue repair by suppressing effector T cells, promoting Treg cells, inhibiting dendritic cell maturation and limiting B cell and NK cell activity [17,20,21,22,23,24]. MSCs also modulate neutrophil and macrophage polarization, favoring a shift toward anti-inflammatory pro-regenerative phenotypes [25,26,27]. In turn, macrophages and T cells can influence MSC function, although the underlying mechanisms remain under active investigation. Among the various MSC sources investigated for regenerative medicine, hAM-derived MSCs have attracted particular interest because of their potent immunomodulatory properties and their origin from a tissue that additionally provides bioactive extracellular matrix and acellular derivatives. Comparative studies have shown that hAM-derived MSCs exhibit potent immunosuppressive activity comparable to that of MSCs isolated from other perinatal tissues, as evidenced by their ability to inhibit lymphocyte proliferation and modulate immune responses [28,29]. Beyond these classical MSC-mediated effects, hAM represents a unique source of immunoregulatory activity because its therapeutic potential is not restricted to the cellular compartment but also extends to extracellular matrix-associated and cell-free derivatives, which are discussed in the following sections.

Indeed, the increasing knowledge on the dynamic synergy between stem cells and immune cells in tissue regeneration and the immune system has been recognized as a pivotal component of the stem cell niches with central influence in shaping the inflammation and repair processes [8]. In this sense, immune-centric regenerative strategies are becoming accepted as critical for the improvement of tissue repair and remodeling. Significant advances have recently been made in developing stem cell therapies and bioengineered tissue constructs, using technological breakthroughs such as organoid culture and 3D bioprinting, which have enabled the establishment of complex personalized regenerative approaches [30]. However, since most existing regenerative strategies have not yet been proven safe or clinically effective, integrating immune modulation is expected to accelerate the development of novel regenerative medicine approaches and improve therapeutic outcomes.

Controlling the immune system using immune cell- and/or biomaterial-based strategies is becoming an attractive approach in regenerative medicine aimed at limiting fibrosis and promoting regeneration. In this sense, biomaterial-based immunomodulation can be achieved through biomaterial selection and/or by the delivery of immunomodulatory additives either with pro-inflammatory activity to initiate the healing process or anti-inflammatory activity to assist the resolution of inflammation [31]. Particularly promising are approaches that integrate biomimetic scaffolds containing acellular tissue-derived matrices, which enable the establishment of native-like microenvironments while exerting pro-regenerative and immunomodulatory features. In this context, the use of placental tissue derivatives—with their immense regenerative and immunomodulatory potential—is emphasized as one of the cutting-edge strategies.

## 3. Amniotic Membrane of Human Placenta as a Source of Bioactive Factors

The human placenta is a complex fetomaternal organ that is essential for sustaining fetal development throughout pregnancy. In addition to its classical physiological roles in nutrient exchange, gas transfer, and endocrine regulation [32], modern research in cell biology and tissue engineering has identified the placenta as a rich source of bioactive components with significant therapeutic potential.

**Figure 2:**
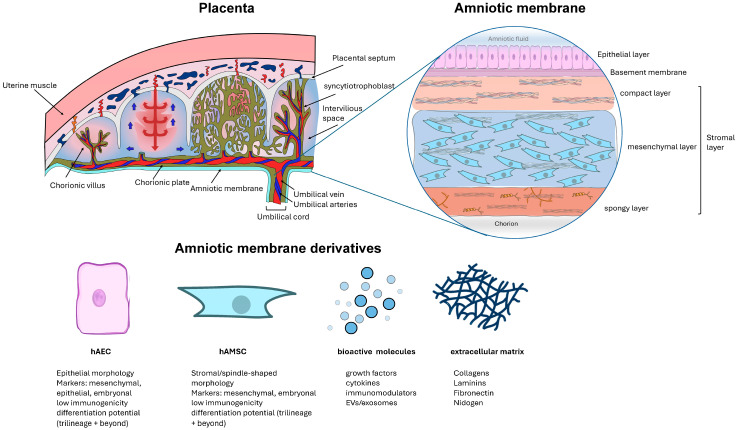
Structural organization and key biological features of the human amniotic membrane (AM). The AM consists of a single epithelial layer overlying basement membrane and stromal layers containing mesenchymal stromal cells. AM-derived cells exhibit low immunogenicity and potent immunomodulatory activity and secrete a broad spectrum of regenerative bioactive factors, including growth factors, cytokines, ECM proteins, and extracellular vesicles. Illustration made with images provided by Servier Medical Art (https://smart.servier.com), licensed under CC BY 4.0 (https://creativecommons.org/licenses/by/4.0/, accessed on 28 June 2026), NIAID NIH BioArt, Bioicons and Scidraw sources.

The placenta comprises both fetal and maternal components—the chorionic villi and decidua basalis, respectively. It is a discoid structure approximately 15–20 cm in diameter and 2–3 cm thick, formed from extra-embryonic tissues, that consists of the placental disc, fetal membranes, and the umbilical cord [33] (Figure 2). The fetal portion includes the chorionic plate and the amniotic and chorionic membranes, which act as a barrier between the fetus and the maternal endometrium [33]. The functional units of the placenta are the chorionic villi, which extend into the intervillous space filled with maternal blood. These villi are covered by a multinucleated continuous layer of syncytiotrophoblast in direct contact with maternal blood and rich in microvilli, which increases the exchange surface area [34]. Beneath it lies a layer of mononuclear cuboidal to columnar cytotrophoblast cells, whose numbers decrease as gestation progresses, leaving the syncytiotrophoblast as the dominant covering layer [34].

The AM is the innermost layer of the placenta; it is avascular and, although very thin, it has a complex multi-layered anatomy (Figure 2). Namely, its epithelial layer surrounding amniotic fluid consists of human amniotic epithelial cells (hAECs) derived from the embryonic epiblast [35,36]. These cuboidal to columnar cells express epithelial markers such as cytokeratin 8/18 and E-cadherin [37,38]. They secrete bioactive factors including TGF-β, hepatocyte growth factor (HGF), and EGF, which contribute to immunomodulation, epithelial repair, and antimicrobial defense through cytokine and antimicrobial peptide secretion [39]. Beneath the epithelium lies one of the thickest basement membranes in human tissues, acting as a mechanical and biochemical interface between the epithelium and mesenchyme. This membrane contains type IV and VII collagen, laminin isoforms (particularly laminin-111 and laminin-511), nidogen, and heparan sulfate proteoglycans, along with bioactive molecules such as tumor necrosis factor (TNF)-α, nerve growth factor, brain-derived neurotrophic factor, and activin [40]. The mesenchymal layer includes a compact sublayer of dense collagen type I and III fibers that confer tensile strength; a fibroblast layer containing spindle-shaped amniotic MSCs (AMSCs) expressing CD73, CD90, and CD105 but lacking hematopoietic markers, which secrete different bioactive molecules; and a spongy layer rich in hyaluronic acid, collagen types III and V, elastin, and proteoglycans, which acts as a viscoelastic cushion, maintains hydration, and modulates inflammation [35,36].

Besides the expression of mesenchymal and lacking hematopoietic cell surface markers, the expression of pluripotency markers and tri-lineage differentiation potential into tissues of mesodermal origin is common to both hAECs and hAMSCs [39,41,42]. These features make them suitable for use in regenerative medicine. Furthermore, the differentiation potential of cellular AM derivatives extends to other tissues, among which are epidermal, hepatic, neuronal and other tissues [43,44,45]. The exact mechanism through which MSCs exhibit their (therapeutic) functions is still not known; however, it is proposed that they mostly do so in a paracrine way. Indeed, AM-derived cells secrete various bioactive molecules, such as aforementioned cytokines and growth factors, but also EVs with diverse cargos that can trigger various effects in targeted tissues, including immunomodulation and regeneration [46,47]. In addition, the ECM of the AM forms a complex three-dimensional scaffold of collagens, laminins, fibronectin, hyaluronic acid, and sulfated proteoglycans, supporting mechanical integrity while influencing cell adhesion, migration, and differentiation. Moreover, hAECs and hAMSCs express very low levels of human leukocyte antigen (HLA)-A, B, C and lack HLA-DR, which makes hAM almost not immunogenic [39,42]. These unique features underpin the placenta’s AM expanding role in regenerative medicine and tissue engineering.

However, evidence indicates substantial variability in the bioactive profile of hAM-derived materials, which represents a major challenge for standardization in biofabrication applications. Both intra- and inter-donor variability have been reported, with growth factors such as b-fibroblast growth factor (FGF), HGF, keratinocyte growth factor (KGF), and TGF-β1 shown to fluctuate depending on donor-specific factors, gestational age, and tissue handling conditions [48]. These findings suggest that the regenerative potential of hAM is inherently heterogeneous, raising concerns regarding batch-to-batch reproducibility. The literature is also not fully consistent regarding the stability of these bioactive components during storage and processing. For instance, a study suggests specific conditions for AM storage (i.e., −80 °C or TC199-enriched medium) and use within 1–7 days after obtaining the tissue to secure the presence of the cytokines [49]. Importantly, the presence of growth factors in preserved hAM should be interpreted with caution as most commonly used preservation methods, including storage at −80 °C, lyophilization, drying, and gamma irradiation, substantially reduce cellular viability [50]. Consequently, the bioactive molecules detected in processed membranes largely represent residual factors retained within the ECM rather than newly synthesized products. Continued secretion of cytokines and growth factors can only be expected when viable cells are preserved, which generally requires controlled-rate cryopreservation with appropriate cryoprotectants and storage at ultra-low temperatures. In this context, Awatara IBNA et al. showed that storage periods of 1–9 months of gamma-irradiated freeze-dried hAM did not adversely affect the levels of EGF, TGF-β, and b-FGF [51]. However, since both lyophilization and gamma irradiation may alter growth factor content and compromise cellular viability, these findings should be interpreted as reflecting the stability of residual matrix-associated factors rather than the preservation of the full biological activity of native hAM. Similar retention of bioactive molecules has been reported following 3D micronization, which preserved basement membrane architecture and detectable levels of NGF, HGF, KGF, b-FGF, TGF-β1, and EGF within the AM matrix [52]. In addition to donor-level variability, spatial heterogeneity within the same membrane has been observed. Although there is a study showing no variation was observed in the expression of HGF, KGF and NGF in different zones of hAM [53], Bischoff M et al. showed that conditioned medium (CM) from AM contained EGF, b-FGF, IL-6 and IL-8, with b-FGF concentration being significantly higher at the central location of the AM in comparison to the peripheral part [54]. Another group demonstrated that EGF was predominantly seen in the AM epithelium at both gene and protein levels; however, its protein content varied inter-donor and at different sites within the same membrane, with the highest concentration in apical and mid-region epithelium [48]. Furthermore, the amounts of EGF, TGF-β and TIMP-1 varied between placental and cervical portions of the same membrane, as well as between physiological at-term delivery and caesarean section-derived membranes, although the authors point out the need for a larger sample group [55]. In addition to donor-level variability, spatial heterogeneity within the same membrane further complicates the assumption of uniform bioactivity within processed hAM-derived preparations.

Collectively, these findings highlight substantial biological and technical variability among hAM preparations. Such heterogeneity may influence the concentration and activity of bioactive molecules, thereby complicating comparisons between studies and potentially contributing to variability in therapeutic outcomes.

### 3.1. Immunomodulatory Potential of hAM-Derived Biomolecules

Immunomodulatory properties of AM are attributed to AM-derived bioactive molecules that activate signaling pathways in different types of immune cells, which induce changes in their multiple functions. Human AMSCs and hAECs secrete various factors with immunomodulatory properties, such as indoleamine 2,3 dioxygenase (IDO), prostaglandin E2 (PGE2), HGF and others [56]. For instance, IDO is a heme enzyme that plays an important role in immune tolerance by inhibiting the proliferation of effector T cells [57], and PGE2 affects T cell activation, proliferation and differentiation; as such, it has been shown to decrease proliferation, stimulate IL-4 and IL-10 secretion and promote adaptive Tregs [58]. With knowledge of the interplay of regeneration and immunomodulation, the evidence of different immunomodulatory effects of hAM is growing (Table 1). Most of the studies focused their attention on the investigation of the immunomodulatory potential of cells from hAM both in vitro and in vivo. However, some studies demonstrated these effects with the whole AM. Namely, decellularized hAM (dhAM) significantly inhibited M1 polarization in LPS-induced macrophages in vitro by inhibiting Toll-like receptors (TLRs) and the TNF signaling pathway while promoting macrophage M2 polarization [59]. When applied in the subcutaneous implant model in rats, dhAM inhibited the persistence of inflammation and fibrous capsule formation, while promoting M2 macrophage polarization, thereby facilitating tissue regeneration [59]. Although the immunomodulatory effects of dhAM resemble those reported for hAM-derived MSCs, their mechanisms are likely distinct. Unlike MSCs, which actively modulate immune responses through cell–cell interactions and secretion of immunoregulatory mediators, dhAM acts as a bioactive ECM scaffold. Preserved ECM components and matrix-associated bioactive molecules may provide instructive cues that influence macrophage polarization and the local immune microenvironment even in the absence of viable cells [56]. Available data show that MSCs derived from hAM significantly inhibited the proliferation of lymphocytes [28,29,60], which was suggested to be both by direct contact and soluble factors [60]. That is in accordance with experiments that show that the levels of immunomodulatory factors such as HGF, TGF-β, IDO and PGE2 in hAMSCs increased during co-culture with peripheral blood mononuclear cells (PBMCs) [61,62]. Moreover, hAMSCs affected CD8 T cell activation and differentiation by reducing the phosphorylation of key signaling molecules like mTOR and AKT and downregulating receptors such as IL-12Rβ1 and IL-2RA [63]. This mechanism led to reduced STAT4 and STAT5 activation, decreased expression of transcription factors like T-bet and Eomes, and ultimately resulted in impaired differentiation of naive CD8+ T cells [63]. As for hAECs, these cells were also shown to significantly suppress activated human PBMC proliferation [64,65] and inhibit monocyte differentiation to dendritic cells, leading to reduced ability to stimulate T cell proliferation [66]. In that study, the authors observed that hAECs were more effective when cultured in direct contact with monocytes than in a Transwell system. A clinical trial suggested that hAEC infusion could be a promising therapeutic strategy for post-hematopoietic stem cell transplantation (HSCT) graft versus host disease (GVHD) without compromising the graft versus leukemia (GVL) effect [67]. They showed that, in the presence of hAECs, the levels of pro-inflammatory cytokines were reduced while the levels of the anti-inflammatory cytokines were upregulated in humanized mouse model of acute GVHD. They further noticed that hAECs regulated CD4+ subset polarization in a paracrine mode, in which TGF-β and PGE2 were selectively secreted to mediate Treg elevation and Th1/Th17 inhibition, respectively. In addition, transplanted hAECs preserved the GVL effect by inhibiting leukemia cell growth. More intriguingly, hAEC infusion in HSCT patients displayed a potential anti-GVHD effect with no safety concerns and confirmed the immunoregulatory mechanisms in the preclinical study [67].

Moreover, CM of hAM has been investigated as a pool of immunomodulatory bioactive molecules. It was shown for instance that CM of hAM fragments significantly inhibit PBMC proliferation, along with markedly reduced levels of Th1 cells and Th1-related cytokines (IFN-γ and TNF-α) in the supernatants of activated PBMCs [68]. On the contrary, all CMs from both single and pooled donors were able to slightly increase the percentage of Th2 cells. Also, the authors observed that CM from hAM was able to significantly decrease the expression of IFN-γ and granzyme B on CD8 T cells as well as common cytotoxicity markers by CD4 T cells [68]. In addition, this study shows that lyophilization of CM from hAM and hAMSCs does not alter the immunomodulatory properties of CM from both sources, confirming the high immunomodulatory potential of hAM. Furthermore, it was shown that, like hAMSCs, their CMs inhibit stimulated PBMC proliferation [69], suppress B cell proliferation [1] and affect neutrophils [70]. Namely, a study showed that hAMSC-CM inhibited neutrophil extracellular trap (NET) release in a PAD-4-dependent pathway, which was mediated by PGE2 [70]. Furthermore, hAEC secretome reduced the amount of NK cells and increased the M2 macrophages in a dose-dependent manner, which was mediated by soluble factors such as macrophage colony-stimulating factor (M-CSF) and could not be induced by hAEC-derived EVs [71]. The authors further demonstrated that the EVs and proteins released by primary human AEC consisted of constitutively expressed proteins, such as vascular endothelial factor (VEGF), IL-12B, and M-CSF. Nano-sized EVs emerge from most of the cells and carry a wide range of bioactive molecules, including nucleic acids, lipids, and proteins. These nanoparticles participate in intercellular communication and regulate various intracellular biological functions, such as immunomodulation [72]. EVs derived from hAECs (containing HLA-G) were shown to significantly inhibit T cell proliferation regardless of their size [65]. Moreover, human AEC-derived exosomes (AECs Exo) alter anti-inflammatory response by inhibition of T cell proliferation, decrease in neutrophil myeloperoxidase (MPO) activity and enhancement of macrophage phagocytosis properties [73]. Also, Alhomrani M et al. reported that, like hAEC-CM, AECs Exo reduce macrophage infiltration and shift macrophage polarization state toward M2 [74]. These results show and confirm the advances in our understanding of anti-inflammatory response affected by EVs originating from AECs.

**Table 1:** Bioactive molecules of hAM involved in immunomodulation.

Type of hAM Derivative	Bioactive Molecule(s) Detected	Immunomodulatory Effect	Ref.
dhAM	Bioinformatics analyses done	Inhibited M1 macrophage polarization (by suppressing TLR and TNF signaling); promoted M2 polarization; reduced inflammation and fibrosis in vivo in a subcutaneous model in rats	[59]
hAMSCs	N/A	Inhibited alloreactive T-lymphocytes in the mixed lymphocyte reaction	[28,29]
hAMSCs; FN-γ-primed hAMSCs	IL-6, IL-10, PGE_2_, IFN-γ, and TNF-α, IDO; miRNA	Inhibited T lymphocytes; induced M2-like phenotype in monocytes and the increase in IL-10 production	[60]
hAMSCs	HGF, TGF-β, IDO, PGE_2_	Suppressed PBMC proliferation in a dose-dependent manner; decreased their IFN-γ IL-17; increased IL-10 in PBMCs	[61]
hAMSCs	IDO	Suppressed PBMC proliferation	[62]
hAMSCs	N/A	Reduced proliferation of naive and differentiated CD8 T cell subsets; suppressed naive CD8 lymphocyte differentiation by downregulating the phosphorylation of mTOR and AKT	[63]
hAECs	lsEV and ssEV; sHLA-G and sHLA-E	Suppressed activated PBMC proliferation	[65]
hAECs	N/A	Suppressed specific and non-specific T cell proliferation; decreased pro-inflammatory cytokine production and inhibited the activation of stimulated T cells in vitro; suppressed the development of EAE in NOD/Lt mice; reduced T cell responses and production of IL-17A; increase in the number of peripheral T regulatory cells and naïve CD4+ T cells	[64]
hAECs	N/A	Inhibited monocyte differentiation into DCs; inhibited IL-12p70 and TNF-α; increased IL-10 secretion in DC.	[66]
hAECs	TGF-β, PGE_2_	Induced CD4+ T cell polarization (Th1 and Th17 subsets were downregulated and Treg subset was elevated); reduced pro-inflammatory cytokines, upregulated anti-inflammatory cytokines; preserved the GVL effect by inhibiting leukemia cell growth; displayed potential anti-GVHD effect in the preclinical study	[67]
Intact hAM- CM	IFN-γ, TNF-α; IL-4, IL-10	Inhibited PBMC proliferation; decreased Th1 cytokines; increased Th2 cells; suppressed cytotoxic markers in CD8^+^ and CD4^+^ T cells	[68]
hAMSC-CM and encapsulated hAMSCs	SDF1a, Groa, IL-6, IL-8, MCP-1, MIP1-b, HGF and VEGF-A	Inhibited PBMSC proliferation	[69]
hAMSC-CM	N/A	Suppressed B cell proliferation	[1]
hAMSC-CM	PGE_2_	Inhibited NET release by neutrophils via PAD-4-dependent mechanism	[70]
hAEC-CM	M-CSF	Decreases number of NK cells; increased number of M2 macrophages in dose-dependent manner	[71]
hAEC-exosomes	N/A	Polarized and increased macrophage phagocytosis; reduced neutrophil myeloperoxidases; suppressed T cell proliferation; reduced lung inflammation in mice	[73]
hAEC-CM; hAEC-exosomes	-bioinformatics analyses applied	Reduced macrophage infiltration; macrophage polarization toward M2	[74]

CM—conditioned medium; EAE—experimental autoimmune encephalomyelitis; EVs—extracellular vesicles; hAM—human amniotic membrane; dhAM—decellularized hAM; hAMSCs—hAM mesenchymal stromal cells; hAECs—hAM epithelial cells; HGF—hepatocyte growth factor; IDO—indoleamine 2, 3-dioxygenase; IFN-γ—interferon γ; IL—interleukin; lsEVs—large-size EVs; miRNA—micro-RNA; M-CSF—macrophage colony-stimulating factor; MCP1—monocyte chemoattractant protein1; PGE—prostalglandin E2; ssEVs—small-size EVs; TGF—transforming growth factor; TNF-α—tumor necrosis factor α; VEGF—vascular endothelial factor.

### 3.2. Regenerative Potential of hAM-Derived Biomolecules

Amniotic membrane is rich in various growth factors, extracellular matrix components and other bioactive molecules that have a role in tissue regeneration. Key growth factors produced by AM, such as EGF, b-FGF, HGF, and platelet-derived growth factor (PDGF), are involved in different processes, such as angiogenesis, cell proliferation, migration, and differentiation, crucial for wound healing and tissue regeneration. Upon binding to their receptors on target cells, different signaling pathways are triggered, inducing changes in gene regulation, which results in changes in cell behavior, ultimately inducing processes underneath tissue regeneration. Different AM derivatives were shown to stimulate these processes in both in vitro and in vivo studies (Table 2). For instance, it was demonstrated that 3D micronized AM containing nerve growth factor (NGF), HGF, keratinocyte growth factor (KGF), b-FGF, TGF- β1, and EGF induced proliferation of epidermal stem cells (ESCs) in rotary cell culture system and by serving as a scaffold for ESC-induced wound healing upon transplantation in full-thickness skin defects in nude mice [52]. The extracts from different portions of hAM containing EGF, TGF-β and tissue metallopeptidase inhibitor-1 (TIMP-1) stimulated proliferation of keratinocyte and fibroblast cell lines (HaCaT and Wi-38, respectively) and inhibited proliferation of endothelial cell line HECa-10 [55]. Available data show that hAM exhibits its paracrine effects related to tissue regeneration also through other cytokines and bioactive soluble molecules. hAMSCs and derived CMs were shown to inhibit apoptosis of skin cells and promote their proliferation through activating PI3K/AKT signaling pathway mediated by cytokines including PAI-1, C-GSF, periostin, and TIMP-1 [75]. When it comes to investigating the direct effect of CM from AM-derived cells, available data show that CMs from hAECs and hAMSCs increased migration and reduced proliferation and differentiation of human keratinocytes, while levels of the proteins related to wound healing, including CTHRC1, LOXL2 and LGALS1, were significantly higher in hAMSC-CM than hAEC-CM [76]. The authors noticed that LOXL2 significantly enhanced in vitro keratinocyte migration and differentiation by activating the JNK signaling pathway. Moreover, the treatment with hAMSC-CM and LOXL2 significantly accelerated wound healing in the murine skin wound model. The paracrine effect of the hAECs was demonstrated also in a model of ovary regeneration, where CM from hAECs rich in TGF-β1, GDF9, and BMP15 stimulated recovery of chemotherapy-induced damage of the ovaries in mice [77].

Angiogenesis, the process of forming new blood vessels from existing ones, is present in both healthy and pathological states. Although AM itself is avascular, its cells secrete bioactive molecules with angiogenic effects. Fresh and cryopreserved hAM displayed distinct secretory profiles, with preservation affecting the abundance of several angiogenic and immunomodulatory mediators [78]. Interestingly, some investigations suggested that, depending on the side, hAM expresses either anti- or proangiogenic activity. For instance, AM implanted on male rats with dorsal skin removed, when epithelial side down, showed inhibition of angiogenesis, while mesenchymal side down induced higher number of vessel sprouts and their lengths increased [79]. The same group showed that cryopreserved AM maintains those effects, except the levels of IL-8 and TIMP-2 in cryopreserved samples were significantly lower, suggesting the effects are probably attributed to other non-cellular components of hAM as well [80]. However, data show that preservation of the cellular components in cryopreserved AM could enhance its angiogenic effect. Namely, Yi Duan-Arnold et al. showed that CM from cryopreserved hAM containing viable cells induced greater endothelial cell recruitment and tube formation compared with devitalized cryopreserved hAM, which was also supported with significantly higher levels of VEGF, b-FGF and PDGF-BB alone [81]. It was demonstrated that CM from cryopreserved hAM exhibits its antiangiogenic effect on HUVECs via TIMP2 and thrombospondin (pigment epithelium-derived growth factor—PEDF—was highly expressed, and, among proangiogenic factors, the highest was only HGF) [82]. As regards CM obtained from AM-derived cells, it was shown that, while hAEC-CM promoted human endothelial cell migration, CM obtained from hAMSCs promoted endothelial cell proliferation, and both CMs promoted angiogenesis in endothelial cells [39]. Amniotic membrane-derived cells secreted significant amounts of angiogenic factors, including HGF, IGF-1, VEGF, EGF, HB-EGF and b-FGF, although some differences are noticed in their levels between cells [39]. In another study, hAMSCs and derived CMs significantly induced neovascularization in a murine model of hindlimb ischemia in 8-week-old Balb/C nude mice, and the authors demonstrated that hAMSCs secreted multiple proangiogenic mediators, including angiogenin, HGF, EGF, IL-8, MCP-1 and TIMPs, which were associated with enhanced neovascularization in vivo [83]. The CM from hAECs containing TGF-β1, GDF9, and BMP15 promoted angiogenesis in chemotherapy- induced damage of the ovaries and enhanced the tube formation of HUVECs [77]. Grzywocz Z. et al. further demonstrated that hAM-derived cells secrete additional growth factors and cytokine receptors involved in angiogenesis and tissue remodeling, with secretion profiles influenced by cell composition and cell–cell interactions [84]. Conditioned medium from these cells induced intensified migration of HUVECs. The published data suggest that the angiogenic effect of the AM and its derivatives could also depend on the microenvironment. For instance, hAECs cultivated in hypoxia secreted higher levels of several cytokines, including angiogenin (ANG), EGF, IL-6, and monocyte chemoattractant protein (MCP)-1, which were found in infarct and border zones of rat myocardium after treatment with hAECs compared to the tissues of saline-treated rats [85]. When applied in vivo, CM enriched with exosomes from AMSCs cultivated under 2% O_2_ stimulated angiogenesis in a rat whole skin-excision model, the authors conclude probably due to the presence of TFGb in exosomes [86]. Furthermore, gestational diabetes mellitus environment enhances the angiogenic capacity of hAMSCs to form tubes and the expression of FGFR2, serpin family E member 1, TGFBR1 and VEGFA [87]. The microenvironment-dependent nature of hAM activity may partly explain why AM exhibits antiangiogenic properties in ocular applications, where maintenance of corneal avascularity is essential, while promoting angiogenesis in skin wounds, where vascularization is required for effective tissue regeneration. Thus, the balance between pro- and antiangiogenic signals associated with hAM appears to be strongly influenced by local tissue conditions and regenerative demands.

**Table 2:** Bioactive molecules of hAM involved in tissue regeneration.

Type of hAM Derivative	Bioactive Molecule(s) Detected	Regenerative Effect	Ref.
3D micronized hAM	NGF, HGF, KGF, b-FGF, TGF-β1, EGF	Induced proliferation of epidermal stem cells; induced wound healing in full-thickness skin defects in nude mice	[52]
hAM extracts	EGF, TGF-β, TIMP-1	Stimulated proliferation of keratinocyte and fibroblast cell lines; inhibited proliferation of endothelial HECa-10 cells	[55]
hAMSCs and hAMSC- CM	PAI-1, C-GSF, periostin, TIMP-1	Inhibited apoptosis of skin cells; promoted proliferation via PI3K/AKT signaling	[75]
hAEC-CM and hAMSC-CM	CTHRC1, LOXL2, LGALS1, ADAMTS1, C3, and CYR61	Inhibited proliferation and differentiation of keratinocytes; increased keratinocyte migration; hAMSC-CM accelerated wound healing in murine model.	[76]
hAEC-CM	TGF-β1, GDF9, BMP15	Promoted angiogenesis in the injured ovaries and enhanced the tube formation of HUVECs in co-culture system; stimulated recovery of chemotherapy-induced ovarian damage in mice.	[77]
Fresh hAM	N/A	In rats with dorsal skin removed: epithelial side down-inhibition of angiogenesis; mesenchymal side down-induced higher number of vessel sprouts, and their lengths increased	[79]
Cryopreserved hAM	IL-8 and TIMP-2	In rats with dorsal skin removed: epithelial surface down-inhibited the vessel sprouts and capillary elongation; mesenchymal side down-enhanced the vessel length and new capillary formation.	[80]
cryopreserved hAM- CM	VEGF, b-FGF, PDGF-BB	Induced greater endothelial cell recruitment and tube formation compared with devitalized AM	[81]
cryopreserved hAM- CM	VEGF; b-FGF (low), HGF TIMP2, Thrombospondin PEDF	Stimulated HUVEC proliferation; inhibited HUVEC angiogenesis	[82]
hAEC-CM, hAMSC-CM	HGF, IGF-1, VEGF, EGF, HB-EGF, b-FGF	hAEC-CM promoted cell migration and angiogenesis of hAoECs; hAMSC-CM promoted cell proliferation and angiogenesis of hAoECs	[39]
hAMSC-CM	MCP-1, Angiogenin, GCP-2, BDNF, IL-6, EGF, IGF-BP4, TIMP-1, TIMP-2, uPAR, IFG-BP6, ENA78, GRO, GRO-a, IL-6R, STNF-R1, IL-8, and HG	Neovascularization in murine hindlimb ischemia model	[83]
hAECs and hAMSCs	IGFBP-6, MCSF-R, PDGF-AB, FGF-6, IGFBP-4, NT-4, and VEGF-R3	Stimulated HUVEC migration	[84]
hAECs (hypoxia-conditioned)	ANG, EGF, IL-6, MCP-1	Enhanced secreted proangiogenic cytokines in rat model of myocardial infarction	[85]
hAMSC-CM Exosomes (hypoxia conditioned)	GF, EGF, FGF1, TGF-β, and VEGF	Stimulated wound closure and angiogenesis in a rat whole skin-excision model	[86]
hAMSCs (gestational diabetes-conditioned)	FGFR2, SERPINE1, TGFBR1 and VEGFA	Increased proangiogenic capacity	[87]
hAEC-EVs	miRNAs and proteins involved in ECM remodeling, migration, proliferation	Ppromoted the proliferation and migration of human corneal epithelial cells and corneal stem cells in vitro; facilitated corneal re-epithelialization, reduced scar formation, and promoted corneal tissue restoration in rabbits with alkali burns	[88]
hAMSC-derived exosomes	miRNAs related to wound healing	Promoted fibroblast proliferation, migration, and angiogenesis in HUVECs and wound healing in diabetic mice via PI3K-AKT pathway	[89]
hAMSC-derived exosomes	miRNA N-149	Promoted fibroblast proliferation and migration and angiogenesis in HUVECs	[90]
hAMSC-exosomes	lncRNAs (PANTR1, H19, OIP5-AS1, NR2F1-AS1)	Stimulated proliferation, migration, angiogenesis of high-glucose-treated HUVECs and wound closure in diabetic models.	[91]
hAM and hAMSC-derived exosomes	N/A	Antifibrotic activity in LX-2 hepatic cell line by inhibiting proliferation, migration, and profibrotic factors.	[3]

CM—conditioned medium; EGF—epidermal growth factor; EVs—extracellular vesicles; FGF—fibroblast growth factor; hAM—human amniotic membrane; hAMSCs—hAM mesenchymal stromal cells; hAECs—hAM epithelial cells; hAoECs—human aorta endothelial cells; HGF—hepatocyte growth factor; IL—interleukin; KGF—keratinocyte growth factor; LncRNAs—long non-coding RNAs; MCP1—monocyte chemoattractant protein1; miRNA—micro-RNA; NGF—nerve growth factor; TGF—transforming growth factor, VEGF—vascular endothelial factor.

Extracellular vesicles, equally important in AM-derived cells’ secretome, have an important role in hAM-induced tissue repair by delivering their cargo to trigger processes underneath tissue regeneration. For instance, hAEC-EVs containing proteins involved in ECM remodeling, migration and proliferation facilitated re-epithelialization of the cornea after alkali burns in rabbits, reduced scar formation, promoted the restoration of corneal tissue and promoted the proliferation and migration of human corneal epithelial cells (hCECs) and human corneal stem cells (hCSCs) in vitro [88]. Exosomes from hAECs rich with wound healing-related miRNAs promoted proliferation and migration of human fibroblasts, facilitated angiogenic activity of HUVECs in vitro and the wound healing process in diabetic mice via the PI3K-AKT pathway [89]. Other studies demonstrated the stimulation of HUVECs also by exosomes isolated from hAMSCs. This treatment with hAMSC-exosomes significantly increased cell proliferation, migration, and angiogenesis of HUVECs by delivering miRNA N-149 [90] or lncRNAs related to angiogenesis, including PANTR1, H19, OIP5-AS1 and NR2F1-AS [91]. The latter also demonstrated wound closure and angiogenesis in diabetic wounds [91]. Furthermore, exosomes isolated from both hAM and hAMSCs exhibited antifibrotic activity on LX-2 hepatic cell line through inhibiting proliferation and reducing migration and profibrotic factors [3]. In addition to all the above, ECM from hAM not only supports different cells as a scaffolding but also by presence of bioactive molecules such as growth factors. Nevertheless, extensive research has been done to demonstrate the regenerative potential of AM derivatives and underlying paracrine mechanisms to better understand how to apply them in tissue engineering.

Importantly, substantial differences in cytokine composition and biological activity have been reported between native tissue, isolated cell populations, conditioned media, extracellular vesicles, and preserved hAM preparations. Such variability complicates direct comparison between studies and highlights the need for greater standardization of hAM-derived products.

## 4. Amniotic Membrane in Regenerative Applications

**Figure 3:**
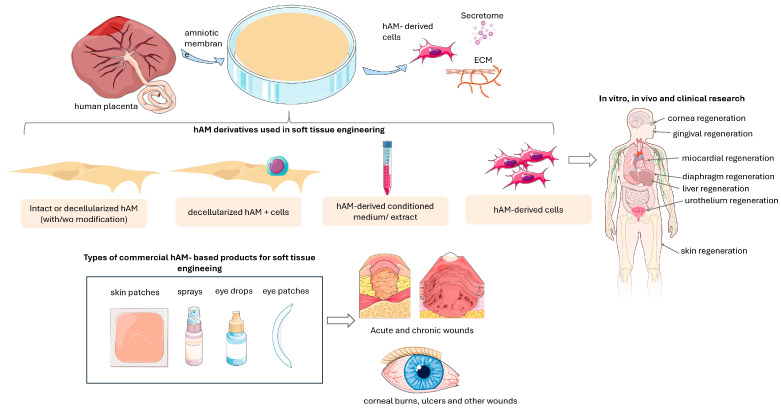
Amniotic membrane derivatives and their applications in soft tissue engineering. The amniotic membrane possesses key regenerative and immunomodulatory properties that support soft tissue repair. These biological features enable a range of AM formats, including fresh, cryopreserved, and decellularized membranes, conditioned medium/extracts, and cells that are applied across diverse soft tissue engineering settings, such as skin and oral wound healing, corneal surface repair, musculoskeletal and tendon regeneration, nerve protection, and soft tissue reconstruction. Illustration made with images provided by Servier Medical Art (https://smart.servier.com), licensed under CC BY 4.0 (https://creativecommons.org/licenses/by/4.0/, accessed on 28 June 2026) and NIAID NIH BioArt Source.

Given the complexity and sensitivity of soft tissues such as tendons, skin, adipose tissue, eye tissue, and other tissues, the engineering of these tissues is a challenging process that requires interdisciplinary knowledge in biology, cell therapy, materials science, and engineering technologies. Relying on the known favorable properties of hAM, its derivatives such as cells, their CM, extracellular vesicles and bioactive molecules have found application in different areas of regenerative medicine, but mostly in dermatology and ophthalmology (Figure 3).

Human AM has been shown to have significant therapeutic potential in the treatment of both acute and chronic wounds as either intact or decellularized, alone or in combination with other cells, or modified/engineered. It was demonstrated that human acellular AM implantation promoted wound healing in Sprague-Dawley rats with full-thickness skin defects [92] and contributed to wound healing and reduced the complication rate in the reconstruction of the lower third of the nose in human patients [93]. Very often, acellular hAM is treated or engineered in order to enhance its effects. For instance, freeze-dried irradiated hAM has demonstrated significant results in buccal fat pad reconstruction [94], while cryopreserved hAM gave excellent results in terms of faster re-epithelialization of gingiva in patients, without signs of infection and the need for analgesics [95]. In comparison to intact hAM, engineered hAM often showed to be more efficient. Human AM coated with electrospun silk fibroin nanofibers achieved better epidermal and dermal regeneration in mice with full-thickness wounds than intact hAM, [96], which was also the case with bioengineered microporous 3D hAM-scaffold in healing of diabetic wounds in rats [97]. Moreover, to stimulate regeneration of wounded tissues many researchers use hAM seeded with MSCs from perinatal tissues or other sources. For instance, in chronic ulcers of diabetic patients, researchers used an acellular hAM scaffold seeded with co-cultured dermal fibroblasts and Wharton’s jelly-derived MSCs (WJ-MSCs) [98]. The authors showed that, within three days, there was significant healing of the wound, and, on the ninth day, complete re-epithelialization was achieved, with no adverse reactions or any complications. Another group demonstrated that combination of WJ-MSCs and dhAM scaffold exhibited significantly better wound-healing capabilities, having reduced scar formation with hair growth and improved biomechanical properties of regeneration in the skin injury of SCID mice model [99]. Decellularized hAM seeded with bone marrow MSCs (BM-MSCs) increased the rate of wound healing in rats with burn wound [100].

Clinical use of hAM has also been found in dermatology and surgery to treat chronic wounds in the form of commercially available hAM-based products. In contrast to Mepitel, a standard wound dressing, dried amnion (AmiCare) and acellular amnion (OcuReg-A) demonstrated significantly greater efficacy in treating split-thickness donor sites [101]. Currently, placental derivatives on the market also consist of dehydrated amnion/chorion membrane allografts (dHACMs), used in treating diabetic foot ulcers, venous ulcers, burns, and surgical defects. Among them is EpiFix, which Zelen et al. demonstrated to be more significantly effective compared to Apligraf, a product containing human skin cells (fibroblasts and keratinocytes) cultured within a bovine collagen matrix, in the treatment of diabetic ulcers of the lower extremities [102]. Another hAM-based product called Grafix contains viable cells and was developed to treat chronic wounds, burns, and postoperative wounds and is more effective but also immunologically less safe [103,104]. Interestingly, Smiell J.M et al. conducted a multicenter observational study on human subjects evaluating wound closure following treatment of uninfected full- and partial-thickness wounds with a decellularized dehydrated hAM (ddhAM) [105]. Notably, 50% of patients who had previously failed treatment with one or more advanced biologic therapies achieved complete wound closure after being treated with ddhAM.

When it comes to the cellular derivatives of hAM, many studies explored the potential of both epithelial and MSCs in treatment of wounds in vivo. For example, topically applied hAECs as well as their CMs improved epithelialization of burn wounds in guinea pigs [106]. When applied in Splint model of diabetic mice with full-thickness wounds, intradermally injected hAECs exhibited high engraftment rates, significantly accelerated wound healing and increased cellularity and re-epithelialization through the secretion of epithelialization growth factors [107]. In a similar way hAMSCs can support wound healing in diabetic mouse wound model [108] and significantly improve blood flow recovery in the hindlimb ischemia model [83]. Human AMSCs and their CMs can also promote thermal burn wound healing in mice by enhancing proliferation, angiogenesis, and inhibiting apoptosis of skin cells in the wound area [75]. Some groups investigated the topical application of hAMSCs with different carriers, and one study showed that, when applied in full-thickness mouse model in combination with Matriderm, they promoted neovascularization and wound closure [109]. In addition, topically applied CM from hAECs significantly accelerated the rate of wound healing in mice with full-thickness excisional skin wound and the formation of skin appendages such as hair follicle and sebaceous gland [110]. The authors suggest this process is mediated with ERK, JNK and AKT signaling pathways. In addition, studies including human patients have shown that the application of human AMSCs promotes skin and soft tissue repair in uremic calciphylaxis, and that, by inhibiting vascular calcification, stimulating angiogenesis and myogenesis, it leads to re-epithelialization and restoration of integrity [111].

In ophthalmology, hAM is used in the reconstruction of the ocular surface [112] for surgical treatment of limbal stem cell deficiency [113] or neurotrophic corneal ulcers [114] and acute ocular chemical burns [115]. Depending on the pathology it can be applied as a graft, patch or eye drops. Cryopreserved hAM was applied with the epithelium facing upward to promote corneal re-epithelialization in refractory neurotrophic corneal ulcers in patients, resulting in significant improvement in pain and complete epithelial closure (60%) in many patients [116]. Wong et al. demonstrated that sandwich transplantation, which consists of amnion/conjunctival-limbal autograft/amnion, is an effective and safe surgical approach for treating recurrent pterygium on eye associated with restrictive strabismus [117]. In a study by Catiglia D. et al., AM-based eye drop formulation successfully restored keratinocyte adhesion in vitro, offering a simple non-invasive therapeutic option for treating corneal lesions in patients with junctional epidermolysis bullosa [118].

Amniotic membrane derivatives were not only used in wound healing but have also shown significant potential in systemic regenerative medicine. Namely, after myocardial infarction, hAMSCs have been shown to contribute to better heart muscle function and structural recovery [119,120], while Weber B. et al. have shown potential in the regeneration of diaphragm muscle tissue, which further confirmed their potential in the treatment of musculoskeletal disorders [121]. In addition, CM from human AMSCs was shown to improve repair after acute myocardial infarction in rats [122] and also holds therapeutic potential in protecting against scar formation. The significance of hAM in regeneration of soft tissues has been demonstrated even more broadly—in the treatment of venous leg ulcers, tendon damage [123], urethral defects [124,125], metabolic liver disorders and acute liver failure and liver fibrosis [126].

The regenerative and immunomodulatory properties of hAM-derived biomaterials discussed in the manuscript have prompted increasing interest in their incorporation into advanced tissue engineering platforms. Among these, 3D bioprinting has emerged as a promising approach for generating biomimetic constructs with precise spatial organization and controlled biological functionality. The combination of ECM components and bioactive signaling molecules present in hAM makes it particularly attractive for biofabrication strategies aimed at enhancing tissue regeneration.

## 5. 3D Bioprinting in Tissue Engineering

**Figure 4:**
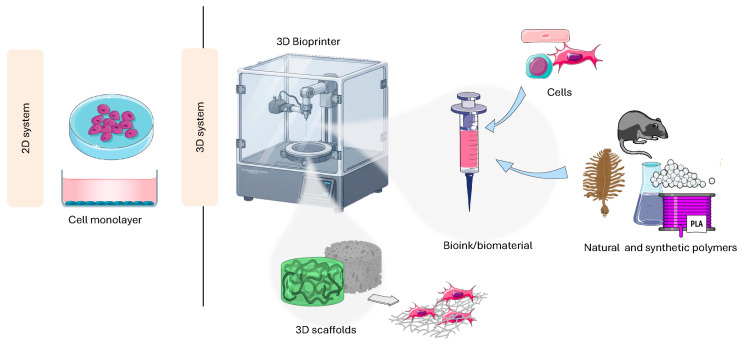
Comparison of traditional 2D cell culture with 3D bioprinting approaches. Schematic representation of conventional 2D monolayer culture (**left**), where cells grow on flat plastic substrates with limited spatial and matrix interactions, compared with 3D bioprinting (**right**), in which cells and biomaterials are combined into bioinks and deposited in a layer-by-layer manner to create structured tissue-like constructs. It illustrates the simplified physiologically limited cell environment in 2D versus the multicellular matrix-supported microenvironment achieved within bioprinted 3D scaffolds that better mimic the in vivo microenvironment. Illustration made with images provided by Servier Medical Art (https://smart.servier.com), licensed under CC BY 4.0 (https://creativecommons.org/licenses/by/4.0/, accessed on 28 June 2026) and Bioicons Source.

Relying on “tissue-engineering triad”, which implies cells, scaffolds, and bioactive molecules, this field uses various techniques to repair or replace damaged tissues. For instance, scaffolds, structural supports for cell growth, are being made by electrospinning, solvent casting, phase separation and other methods. As a novel technique of additive manufacturing in tissue engineering, 3D bioprinting aims, among other things, to replace standard 2D cell culturing methods (Figure 4). 3D bioprinted constructs offer a range of advantages over 2D cell culturing methods, such as being able to mimic tissue structures, to provide the ideal templates for cell proliferation similar to the native tissue, and to be fine-tuned to replicate the mechanical properties of the targeted tissue, which also makes them a suitable tool for drug delivery and toxicity testing [127]. Unlike 3D printed constructs, the 2D cell culture methods represent monolayers of cells without being able to form vascular networks or replicate on-demand structures. Although simpler, cheaper and easily reproducible, the 2D cell culture methods lack wider application and personalization [128], while 3D bioprinting has found its place in personalized regenerative medicine, where tissue-derived cells may be used for creating personalized implants with patient-specific geometry obtained by CT, MRI or other methods [129].

A 3D printed scaffold represents an elevated form of 2D printing since layers of printed bioink are stacked to obtain the desired form and should imitate the cellular environment and the ECM [130]. In order to consider a 3D bioprinted scaffold for soft tissue engineering purposes, biomechanical properties must be investigated. These properties include biocompatibility of bioink (usually defined as a mixture of biocompatible materials and cells), material porosity and swelling, printability, mechanical properties (rheological, compressive, and tensile properties) and degradation kinetics, as well as cellular compatibility [131,132,133]. After 3D bioprinting, the scaffolds are further crosslinked if needed and subjected to further testing with different cell lines [134]. Different tissues and applications require different materials in the production of biocompatible scaffolds. Depending on the desired final product, several different techniques may be used in order to produce a satisfactory scaffold for the same tissue. The most commonly used 3D bioprinting techniques today are extrusion-based bioprinting, droplet-based bioprinting and photocuring-based bioprinting [135]. Each of these basic techniques has its subcategories depending on the mechanism and methodology of bioprinting. Due to the fact that the application of 3D bioprinting has evolved drastically in recent years, some of the subcategories may be found as part of other techniques. Constant improvement of available technology, software and development of specifically designed apparatus may contribute to further complications in precise categorization of 3D techniques.

To date, soft tissue engineering has used many different cells and natural and synthetic materials to produce the best possible bioink. Natural and synthetic materials have been used either as the main bioink constituents or as a combination of several different materials. Natural materials are derived from animals (collagen, fibrin, gelatin, and hyaluronic acid) or plants (alginate, agar, and cellulose), while synthetic materials (PVA, PEG, and polycaprolactone) are obtained through various chemistry techniques. Cells are usually seeded on the printed scaffolds or mixed into the previously formulated bioink and then printed depending on the experimental setting and desired clinical application. Many primary and/or stem cells (including those from AM) are used for this purpose, in addition to endothelial cells commonly used to follow the angiogenic effect, such as human umbilical vein endothelial cells (HUVECs), human microvascular endothelial cells, and induced pluripotent stem cell-derived endothelial cells (iPSC-ECs) [136]. In fact, available data show various combinations of cells and biomaterials used for 3D bioprinting of soft tissues (Table 3). For instance, GelMA–gelatin (crosslinked gelatine with MA) bioink loaded with HUVECs was used for production of vascularized scaffolds for soft tissue engineering [137], or in combination with MC/MCMA loaded with hADSCs, HUVECs and chondrocytes for production of bioink for 3D bioprinting at room temperature [138], or with methacryloyl hyaluronic acid and adipose tissue dECM pre-gel loaded with hADSCs for skin regeneration [139]. Chiesa et al. have explored the potential application of gelatin-nanohydroxyapatite (gel-nHA) for 3D bioprinting of vascular bone model scaffold. The gel-nHA scaffolds were seeded with hMSCs and HUVECs one after another, in a time span of 14 days, thus resulting in bone vascularization [140]. Interestingly, combination of more than two biomaterials together with cells showed impressive results. For example, immortalized megakaryocyte progenitor cell line (imMKCL) was used with silk fibroin–type A gelatin–alginate bioink for 3D bioprinting of human blood progenitor cells [141], while growth, metabolic activity, and structural organization of four mammalian cell lines (HEK, MDCK, CHO, and Vero) were monitored in 3D bioprinted constructs developed by mixing natural materials, such as fibrinogen, alginate, and gelatin [142]. Furthermore, human 3D corneal models were successfully bioprinted with bioinks containing agarose–collagen biomaterial and corneal stromal keratocytes, which maintained their native keratocyte phenotypes for 7 days [143]. Polycaprolactone (PCL), is often used for 3D bioprinting as it is a biodegradable, biocompatible and bioresorbable synthetic material. Namely, PCL scaffolds produced by an electrohydrodynamic cryoprinting method showed a rock-like surface structure, improving adipose-derived MSC cell adhesion and further improving wound healing in rat models [144]. This material is also used in different formulations. Namely, PCL/graphene oxide scaffolds coated with gelatin/CuO nanoparticles seeded with H9C2 cell line demonstrated great potential for future myocardial tissue engineering [145], while 3D bioprinted PCL/collagen dermal scaffolds loaded with human dermal fibroblasts and epithelial keratinocytes successfully fabricated full-thickness biomimetic skin equivalent [146]. Furthermore, porcine aortic valve interstitial cells (PAVICs) seeded on poly-ethylene glycol-diacrylate (PEGDA) 3D printed aortic valve retained almost 100% of their viability over the course of 21 days [147].

Fast progress in tech development has led to many upgrades and modifications to the original 3D printing methods and machines. Moreover, many research groups have proposed and developed in-house printing solutions based on existing printers, such as Freeform Reversible Embedding of Suspended Hydrogels (FRESH) bioprinting. Nevertheless, the implementation of 3D techniques spans beyond tissue engineering across drug delivery and screening [148], disease modeling [149,150], organ on-a-chip [151] and organoid production [152,153], orthodontic and orthopedic implants [153,154,155], printing food [156] and other fields.

### hAM Incorporation into 3D Biofabricated Constructs: Current Applications and Limitations

A bioink is proposed to be defined as a formulation of cells suitable for processing by an automated biofabrication technology that may also contain biologically active components and biomaterials to distinguish from biomaterial inks that are (bio-)materials that can be printed and subsequently seeded with cells after printing [157]. As biomaterials should provide biocompatible components, favorable rheological properties, and support living cells in terms of their functions and 3D environment, such as ECM, hAM represents a good starting material for formulating biomaterial inks. As stated earlier in this review, hAM is a natural tissue considered as medical waste whose collection is not invasive; it has regenerative and immunomodulatory features; it consists of cells with stem cell features, extracellular matrix and different bioactive molecules and is minimally immunogenic.

The fields of tissue engineering and biofabrication have been exploring various methods for the use of hAM for regeneration of different tissues, offering several strategies that could be used in 3D bioprinting [158]. For instance, dhAM could be used as printable ECM scaffolds for cells. Also, hAM could be printed as a hydrogel delivery system, or cells and CM and extract derived from hAM could be used as additives to bioinks. Available data suggests that, among all hAM derivatives, dhAM gained the most attention in recent years in the field of tissue engineering and regenerative medicine, mostly in wound healing. Indeed, over the years, various decellularized ECMs derived from different tissues have been investigated as bioinks for 3D bioprinting applications [159]. Namely, several approaches of hAM decellularization have been proposed [160], producing dhAM derivatives that are used either alone or mixed with natural or synthetic materials in order to create AM composites. In addition, hAM has also been applied in wound healing in different preparations—powdered AM, coated AM, AM extract and AM-based hydrogels [161].

The currently available literature on hAM incorporation into 3D constructs remains limited and spans diverse regenerative applications rather than exclusively soft tissue contexts. Therefore, this subsection includes representative studies from cartilage-, bone-, and soft tissue-related models to provide a comprehensive overview of the emerging use of hAM-derived components in biofabrication and bioprinting approaches. These studies mostly focused on dhAM in combination with different compounds, and, to the best of our knowledge, only one using hAMSCs (Table 4). One such study developed a bioink based on dhAM combined with alginate to directly print a 3D cell-laden perivascular construct that enhanced cell viability, proliferation, tube formation, and expression of VEGFR-2. They compared different combinations of alginate and powdered dhAM selected based on the optimal viscosity for coaxial extrusion bioprinting. To print out the 3D cell-laden vasculature networks using the optimized bioink encapsulated with HUVECs, both bioink and CaCl2 were used through the sheath and core nozzle, respectively. For this research, powdered dhAM was used to preserve native ECM and biochemical components while enabling slow crosslinking. The authors noticed that hAM-based bioinks exhibited good printability without any coagulation or nozzle blockage and that crosslinking was successful and instant. Also, the printed constructs did not need any support and maintained their shape fidelity and perfusability when the second hydrogel was added to generate the 3D revascularized constructs [162]. When it comes to use of natural polymers in 3D bioprinting, shape fidelity, mechanical durability issues and rheological behavior are listed as difficulties. Therefore, different strategies are being used to overcome these issues, including physical and chemical modifications of the bioinks. A study by Kafili et al. demonstrated that adding Laponite nanoparticles to composite sodium alginate-dAM hydrogel improves stability and printability of the formulated biomaterial while maintaining biocompatibility with skin cells [163]. Namely, to obtain modified biomaterial, this research group mixed dhAM–alginate solution with Laponite–alginate solution with different concentrations of Laponite that were followed by neutralization with NaOH. It was noticed that bioinks containing 2% Laponite form aggregates, probably due to the interaction between charged nanoplatelets with ions of dAM solution, causing nozzle clogging and discontinuity of the printing pattern. However, modification of the biomaterials with 1% Laponite enhanced the dynamic mechanical modulus of the hydrogel up to 16-fold, which yielded freestanding and stable constructs with high-dimensional precision and accuracy and finer pore structure. Importantly, these 3D bioprinted constructs enhanced the migration of human fibroblasts in vitro. The same research group further investigated the effects of adding Laponite to hydroxyethyl cellulose (HEC) dhAM-based biomaterials on their printability [164]. They noticed that extrusion printing of bare dhAM biomaterial was not feasible due to low viscosity, while the addition of HEC as a thickening agent provided structural stability. Incorporating Laponite into the biomaterial significantly decreased the spreading ratio, leading to better printability due to crosslinking effect of the Laponite. Moreover, the extracts from the HEC-dhAM biomaterials containing Laponite improved proliferation and migration of human fibroblasts compared to those without Laponite. These findings highlight a recurring challenge in hAM-based biofabrication. While hAM-derived matrices provide biological cues supportive of cell viability and tissue regeneration, their rheological and mechanical properties are often insufficient for standalone bioprinting, necessitating formulation strategies that balance bioactivity with printability. The same group also tested the impact of porcine dAM concentration on the printability and rheological properties of biomaterials solely made from AM by using different AM concentrations and extrusion bioprinting parameters [165]. The results showed that 3% *w*/*v* dAM exhibited the best shape fidelity, while 2% *w*/*v* provided the best balance between printability and cell performance, and all the groups showed high cell viability, with 3% reducing fibroblast proliferation. The authors concluded that 2% *w*/*v* porcine dAM provides proper shape fidelity of printed flat patterns and a supportive microenvironment for fibroblasts. However, a general limitation was the collapse of 3D printed constructs due to slow gelation. In another study, a bioink containing HUVECs and human skin fibroblasts was formulated by methacrylation of the ECM extracted from dhAM and associated with methacrylated hyaluronic acid [166]. Successful fabrication of stable 3D structures with microporosity that contain and support HUVECs was achieved by microextrusion 3D bioprinting and UV crosslinking. Interestingly, the authors were able to replicate the results when applying microvalve and laser-assisted bioprinting. Moreover, they showed successful support of bioprinted HUVECs in co-culture with fibroblasts, which remained viable and were able to organize into capillary-like networks after 14 days, promoting optimal vasculogenesis. On the other hand, by adding hAM extract (AME) to gelatin-methacryloyl (GelMA)–gelatin biomaterial, the printed bioscaffold gained proangiogenic effects, demonstrated by the support of HUVEC proliferation [167]. This research aimed to create a two-layer bioprinted scaffold simulating epidermis and dermis by extrusion bioprinting. To print the scaffold mimicking the epidermis, GelMA/gelatin containing keratinocytes was used, while GelMA/gelatin supplemented with AME and containing fibroblasts and HUVECs was used for the dermis. The separate printing of the structures was performed at a temperature maintained at 4 °C followed by UV crosslinking and joining together by adhesive characteristics of GelMA. Besides increase in number and viability of HUVECs, the AME component of the bioink enhanced the gene expression of the VEGF in these cells. In a study by Hashemi-Afzal F et al. a bioink composed of dhAM, gellan gum (GG), oxidized GG (OGG), aminolyzed polycaprolactone nanofibers (A-PCL NFs) and rat BMSCs was printed into scaffolds [168]. Crosslinking was achieved with CaCl_2_ in addition to the Schiff base imine formation between aldehyde groups of OGG and amine groups (dHAM and A-PCL NFs). The biomaterial composition design incorporated A-PCL NFs to enhance mechanical integrity and higher concentrations of dhAM to boost bioactivity. Indeed, the authors reported that bioinks exhibited excellent printability, forming uniform grids with high shape fidelity and interconnected porosity suitable for cell infiltration and nutrient transport. Moreover, the printed constructs exhibited appropriate viscoelastic recovery and hydration-mediated swelling. The incorporation of A-PCL NFs and increasing dhAM content in composites improved structural stability, shear resistance and low swelling ratio due to dhAM contribution to mechanical reinforcement through additional Schiff base crosslinking sites. The synergistic effect of bioactive dhAM components and the mechanical reinforcement provided by NFs further enhanced the viability of the cells in bioink. The rBMSCs were uniformly distributed throughout the scaffolds, strongly adhered to the matrix and successfully differentiated into chondrocytes, indicating the scaffolds’ potential to stimulate cartilage differentiation.

According to the available data, a couple of studies extended their research on hAM-based biomaterials and bioinks to in vivo applications. For instance, Sarkar et al. explored the application of 3D bioprinted hAM-based constructs for treating partial and full-thickness infected wounds and burns in vivo [169]. They formulated a biomaterial combining lyophilized and micronized dhAM with a solution containing A-type skin gelatin and hyaluronic acid while using transglutaminase as a crosslinking agent. The hAM-based biomaterial was then subjected to extrusion bioprinting with alternating cycles of high (100%) and mild (0.1%) strains at a constant frequency. The constructs demonstrated mechanical resilience through shape recovery after cyclic straining and effectively supported growth of human dermal fibroblasts. To create a wound healing dressing, hAM-based biomaterials were 3D printed over the in-house-developed silver impregnated laminated polyurethane foam. This 3D printed hybrid wound dressing showed comparable wound closure rates and significant bacterial reduction when applied on Wistar male rats with full-thickness wounds. The authors also noticed that, compared to hAM powder only, the dhAM-printed wound dressing increased CD31 expression and improved neovascularization, with peak effects on day 10. Another group developed a 3D printed scaffold based on hydroxyapatite/bone morphogenetic protein-2–mineralized dAM (HAMMA/HAP/BMP-2) for orbital defect repair, addressing the key challenges in orbital reconstruction [170]. For that purpose, they prepared the biomaterial by grafting methacrylic anhydride (MA) onto dhAM, which was mineralized with a HAP/BMP-2 coating, and the addition of HAP particles enhanced the mechanical strength of the hydrogel, increasing the elastic modulus while exhibiting favorable tensile and bending resistance and a reduced degradation rate. Furthermore, these composite HAMMA/HAP/BMP-2 3D printed scaffolds released BMP-2 in a continuous manner in vitro, consistently enhancing the activity of the murine preosteoblast cell line over time, as evidenced by enhanced migration, ALP activity, mineralization and expression of osteogenic factors. The stimulative effects of these scaffolds were confirmed in vivo in a rat skull defect model and canine orbital wall bone defect model, where the degradation of composite scaffolds coincided with the ingrowth of new bone tissues. Collectively, these studies demonstrate that dhAM can improve the biological performance of bioinks; however, most formulations still require combination with carrier polymers, rheological modifiers, or reinforcing agents to achieve adequate printability and structural stability. Thus, despite its favorable biological properties, dhAM alone rarely fulfills all the requirements of an optimal bioink.

Most studies rely on dhAM rather than viable cells. However, one publication reported the use of hAMSCs within a bioink for 3D bioprinting [171]. Feng M. et al. investigated the bioink based on methacrylated gelatin and collagen (GelMA/ColMA) hydrogels loaded with hAMSCs to print scaffolds to treat intrauterine adhesion (IUA), leveraging the evidence that these cells support endometrium repair in rat models. This research showed that the cells within these porous scaffolds, with improved mechanical properties and prolonged degradability, remained alive after the printing and were continuously released for at least 7 days in vitro, showing normal morphology with high proliferation rates. Moreover, application of the GelMA/ColMA/hAMSC scaffolds in the rat model of IUA resulted in decreased fibrotic area and loss of atrophy of the endometrium, confirming their therapeutic repair effect.

To the best of our knowledge, these are the only papers published on 3D bioprinting by using hAM, with none exploring its immunomodulation so far. Although there are still not many studies that investigate the use of hAM for 3D bioprinting, these data confirm that dhAM, its ECM and extract could be used as starting materials for this approach with preserved angiogenic and regenerative potential. Taken together, the properties discussed throughout this review indicate that hAM-derived materials offer a distinctive combination of structural and biological characteristics compared with other regenerative biomaterials. Similar to collagen- and decellularized ECM-based scaffolds, hAM provides a native ECM that is rich in collagens, glycoproteins, and other bioactive components that support cell adhesion and tissue regeneration. However, unlike purified collagen biomaterials, hAM also contains a broad repertoire of growth factors, cytokines, and EVs that contribute to its immunomodulatory and regenerative effects. In this regard, hAM shares certain characteristics with MSC secretomes, which are primarily valued for their biological activity and paracrine signaling. Nevertheless, hAM uniquely integrates structural ECM support with endogenous bioactive signaling molecules within a single readily available and low-immunogenicity tissue source. A comparative overview of these regenerative platforms is presented in Table 5.

## 6. Challenges and Future Perspectives

In this review, we explored the potential of hAM-derived materials as biomaterials for 3D bioprinting-based tissue engineering, emphasizing the interplay between regenerative and immunomodulatory functions mediated by hAM derivatives. Although hAM has been extensively investigated in conventional tissue engineering, its integration with advanced 3D bioprinting technologies remains largely unexplored. The available literature indicates that hAM derivatives hold considerable promise as bioactive components in biofabrication strategies, offering a natural, ethically acceptable and immunomodulatory-rich biomaterial platform. To date, studies incorporating hAM-derived materials into 3D bioprinted constructs have primarily focused on cell viability, differentiation, tissue regeneration, and mechanical performance, whereas the immunomodulatory properties of hAM within bioprinted systems remain largely unexplored. Future investigations could address this gap through the development of bioinks containing decellularized hAM ECMs, hAM-derived EVs, CMs, or purified immunomodulatory factors. Such constructs could be evaluated using co-culture systems involving immune cell populations to assess cytokine production, inflammatory signaling pathways, and macrophage polarization. Furthermore, advanced bioprinting approaches may enable spatially controlled distribution of hAM-derived factors within engineered tissues, allowing researchers to investigate how localized immunomodulation influences tissue repair and regeneration. These studies could provide valuable insight into the role of hAM-derived bioactive molecules in shaping the immune microenvironment of bioprinted constructs.

Despite growing interest, the translation of hAM-derived materials into standardized printable bioinks remains at an early stage. The physicochemical and rheological properties required for bioink design are insufficiently characterized for many hAM-derived preparations. As a result, most current studies utilize hAM derivatives as biofunctional additives incorporated into established hydrogel systems rather than as standalone bioinks. In addition, the compatibility of hAM-derived formulations with different bioprinting modalities remains incompletely explored, with the majority of studies relying on extrusion-based approaches and a lack of systematic comparisons across printing technologies.

Standardization and reproducibility remain major challenges for clinical translation. These issues are closely associated with regulatory and ethical considerations, including donor screening, tissue procurement, and compliance with tissue banking regulations. Processing, storage, and sterilization procedures must adhere to relevant regulatory frameworks; however, these standards are not yet globally harmonized [172]. Furthermore, together with donor-dependent variability, the absence of standardized protocols for the isolation, characterization, and application of perinatal derivatives, including hAM-derived components, contributes to variability across studies [173]. Establishing quality-control parameters and potency assays may be particularly important for reducing batch-to-batch variability and improving reproducibility across studies and clinical applications. A further limitation is the lack of uniform nomenclature for perinatal tissues and their derivatives, which hinders data comparability and reproducibility [174]. From a translational perspective, scalability is constrained by limited tissue availability and logistical factors related to collection infrastructure, donor consent, and clinical awareness. An additional unresolved question is whether bioactive factor integrity and functional activity are preserved following processing, decellularization, and storage, which may directly affect regenerative performance.

Beyond these challenges, 3D bioprinting itself presents inherent limitations, including high equipment cost, material consumption, optimization complexity, and limited resolution, which is strongly influenced by nozzle size [175]. Therefore, the development of hAM-based bioinks requires further optimization of material composition, crosslinking strategies, and printing parameters. A systematic framework for future studies should focus on tailoring rheological properties, swelling behavior, degradation rates, and crosslinking conditions to match the biomechanical and biochemical characteristics of target tissues. Such standardization would improve reproducibility across studies and support the translation of hAM-based bioprinted constructs into personalized regenerative applications.

### Concluding Remarks

In summary, hAM represents a unique source of bioactive molecules that are capable of simultaneously regulating inflammation and promoting tissue regeneration. Through the coordinated action of growth factors, cytokines, ECM components, and EVs, hAM-derived products may influence both immune responses and tissue repair processes, highlighting the close interplay between immunomodulation and regeneration. Although current applications have largely focused on native membranes, extracts, CMs, or decellularized ECMs, emerging biofabrication technologies offer new opportunities to harness these properties in a more controlled manner. In particular, 3D bioprinting may enable incorporation of hAM-derived bioactive factors into engineered tissues, creating biomimetic constructs that are capable of directing cellular behavior and modulating the local immune microenvironment. While significant challenges remain regarding standardization, preservation of biological activity, and mechanistic understanding, the convergence of hAM-based biomaterials, immunoregulatory strategies, and advanced biofabrication technologies represents a promising avenue toward next-generation regenerative therapies.

## Figures and Tables

**Table 3:** Different biomaterial compositions for 3D bioprinting of soft tissues.

Biomaterial Formulation	Cell Incorporation	Tissue Application	Bioprinting Method	Ref.
GelMA	Cell-laden (HUVECs)	Vascularized soft tissues	Extrusion + coaxial	[137]
(adipose tissue ECM) dECM-GelMA-HAMA	Cell-laden (hADSCs)	Skin	Extrusion-based	[139]
Gelatin-nanohydroxyapatite	Cell-seeded (MSC/HUVECs)	Vascularized bone model	Extrusion (piston-driven) with sacrificial material Pluronic F127	[140]
GelMA, MC-GelMA and MCMA-GelMA	Cell-laden, acellular/cell-laden support bath	Tissue engineering	Extrusion (pneumatic), embedded	[138]
Silk fibroin–alginate–gelatin	Cell-laden (imMKCL)	Platelet differentiation	Extrusion	[141]
Fibrinogen–sodium alginate–gelatin	Cell-laden (HEK, MDCK, CHO, and Vero)	N/A	Extrusion	[142]
Type I collagen, agarose–collagen	Cell-laden (CSKs)	Cornea	DoD (electromagnetic microvalve)	[143]
Polycaprolactone	Cell-seeded (AMSCs)	Cell adhesion structure support for tissue damage	Electrohydrodynamic cryoprinting	[144]
Poly ε-caprolactone/GO coated with gelatin/CuO nanoparticles	Cell-seeded (H9C2 cell line)	Myocardial tissue engineering	Fused deposition modeling	[145]
PCL/collagen	Cell-laden (dermal fibroblasts and epithelial keratinocytes)	Full-thickness skin model	Extrusion	[146]
Poly-ethylene glycol-diacrylate (PEGDA)	Cell-seeded (PAVICs)	Aortic valve	Extrusion	[147]

CSKs—corneal stromal keratinocytes; DoD—drop-on-demand; GO—graphene oxide; hADSCs—human adipose-derived stem cells; HUVECs—human umbilical vein endothelial cells; MKCLs—human iPSC-derived megakaryocytes; PAVICs—porcine aortic valve interstitial cells; PCL—polycaprolactone.

**Table 4:** hAM derivatives as biomaterials for 3D bioprinting.

hAM Derivative	Bioink/Biomaterial Formulation	Bioprinting				Scaffold Characterization	In Vitro/In Vivo Model	Effect	Ref.
		Method	Nozzle Size/Printing Parameters	Size and Shape of the Scaffold	Crosslinking				
dhAM	dhAM + Alginate	Coaxial extrusion-based	Coaxial nozzle: 20 G (ID 0.548 mm) + 16 G (ID 1.19 mm); printing speed 15 mm/s;	3 × 3 × 0.5 cm^3^ one- and four-layer lattice; spiral microchannels	4% CaCl_2_ (flow and post-printing)	Perfusion, printability; FESEM, degradation, tensile test, biocompatibility	Cell-laden (HUVECs)	Enhanced cell viability and proliferation; tubulogenesis	[162]
dhAM	dhAM + Alginate + Laponite nanoparticles	extrusion	23 G nozzle (337 μm ID) pressure: 80 kPa; speed: 4, 6, 8 mm/s	Lattice pattern; cylinder 10 mm × 10 mm	0.5% CaCl_2_ 30 min	Hydrogels: FESEM, FTIR, hydrophilicity, rheology; scaffolds: printability; biocompatibility	Cell-seeded (fibroblasts)	Enhanced migration	[163]
dhAM	dhAM + hydroxyethyl cellulose + Laponite	extrusion	23 G nozzle (337 μm ID) pressure: 80 kPa; speed: 4, 6, 8 mm/s	3 cm × 3 cm lattice pattern	Thermal (37 °C for 1 h)	Biomaterials: FE SEM, FTIR, hydrophilicity, biodegradation, tensile tests, rheology; scaffolds (extracts): biocompatibility	Cell-seeded with hydrogel extracts (fibroblasts)	Promoted migration	[164]
dhAM-derived ECM	Metacrylated ECM+ metacrylated hyaluronic acid	microextrusion	0.21 mm diameter nozzle pressure: 4–5 mPa;	Cylinder: 5 mm diameter, 4 mm hight;	UV light (2 w, 365 nm, 10 s)	SEM, biocompatibility	Cell-laden (HUVECs, HSFs)	Sustained HUVEC viability, proliferation, and maturation, promoting optimal vasculogenesis	[166]
hAM-derived extract	GelMA + hAM extract	extrusion	22 G nozzle; speed: 20 mm/s; temperature: 4 °C	Cube model (10 × 10 × 4.8 mm)	UV light	Tensile test, swelling, degradability, SEM, biocompatability	Cell-laden (keratinocytes, fibroblasts, and HUVECs)	Proangiogenic effect-supported HUVEC proliferation	[167]
dhAM	dhAM + GG, OGG + A-PCL NFs	extrusion	22 G nozzle (410 µm ID); pressure: 1–1.5 bar; speed: 10–15 mm/s; 0.5 mm inter-fiber spacing	Square, four- and six-layer grid	100 mM CaCl2, 10 min	FTIR, rheology, SEM, optical microscopy, swelling behavior, biodegradation analyses	Cell-laden (rBMSCs)	Enhanced cell viability; enhanced chondrogenic differentiation	[168]
Lyophilized dhAM	Lyophilized dhAM + A-type skin gelatin + hyaluronic acid + transglutaminase	extrusion	Nozzle: 0.25 mm ID; retraction distance: 4.5 mm; initial layer thickness: 0.1 mm; initial layer line width: 100%; speed: 20 mm/s; bottom layer speed: 10 mm/s	4 × 4 cm	N/A	Biomaterials: rheology, FESEM and RAMAN; scaffolds: SEM and biocompatability	Cell-seeded (human dermal cell line); rodent *Staphylococcus aureus*-infected wound model	Enhanced wound closure; increased CD31 expression; improved neovascularization in full-thickness infected wounds and burns in vivo	[169]
dhAM	MA + dhAM mineralized with (HAP)/BMP-2	N/A	N/A	N/A	UVLED 395–480 nm, 10.5 mm curing tip; for 30 s	SEM; degradation; swelling; biocompatibility	Cell-seeded (MC3T3-E1); rat skull defect model; canine orbital wall bone defect model	Enhanced migration, ALP activity, mineralization and expression of osteogenic markers in MC3T3-E1 in vitro; ingrowth of new bone tissue in vivo	[170]
hAMSCs	AMSC + GelMA/ColMA	N/A	Speed: 20 mm/s; fiber diameter:100 μm; base plate temperature: 4 °C; nozzle temperature: 20 °C	2- layer lattice structure: 16 mm × 16 mm,	Blue light, 10 s	Scaffolds without hAM; swelling, rheological properties, compression tests, morphology with SEM; hAMSC-laden scaffolds: release effect	Cell-laden (hAMSCs); rat IUA model	Prevented cavity adhesion in a rat IUA model	[171]

A-PCL NFs—aminolyzed polycaprolactone nanofibers; BMP-2—bone morphogenetic protein 2; ColMA—methacrylated collagen; dhAM—decellularized hAM; FESEM—field-emission SEM; FTIR—Fourier transform infrared spectroscopy; GG—gellan gum; GelMA—gelatin-methacryloyl; HSF—human skin fibroblasts; HUVECs—human umbilical vein endothelial cells; HAP—hydroxyapatite; ID—inner diameter; IUA—intrauterine adhesion; MA—methacrylic anhydride; MC3T3-E1—murine preosteoblasts; OGG—oxidized GG; rBMSCs—rat bone marrow-derived MSCs; SEM—scanning electron microscope; UV—ultraviolet.

**Table 5:** Comparison of hAM-derived biomaterials and other regenerative platforms with respect to their structural and biological characteristics.

Feature	hAM-Derived Biomaterials *	Collagen Biomaterials	Decellularized ECM	MSC Secretome
Structural ECM support	Yes	Yes	Yes	No
Native tissue microenvironment	Partially retained	Limited	Yes	No
Growth factor reservoir	Present; processing-dependent	Limited	Variable	High
Immunomodulatory activity	Potentially retained; processing-dependent	Limited	Variable	High
Paracrine bioactive factors/EVs	Partially retained; processing-dependent	No	Limited	Yes
Major advantage	Combines ECM and bioactive components	Simple and widely used	Tissue-specific ECM	Potent cell-free signaling
Major limitation	Donor variability and standardization	Limited bioactivity	Processing complexity	Manufacturing and standardization challenges

Adapted from information discussed throughout this review and supported by Refs. [11,16,17,18,66,139,143,162,163,164,166,167,168,169,170,171]. * The properties listed for hAM-derived biomaterials refer to the biological characteristics of hAM as the source material and its derived products. The extent to which these properties are preserved in hAM-derived biomaterials depends on donor variability, tissue processing, preservation and sterilization methods, and the specific type of derivative used (e.g., intact membrane, decellularized forms, extracts, etc.).

## Data Availability

No new data were created or analyzed in this study. Data sharing is not applicable to this article.

## Abbreviations

The following abbreviations are used in this manuscript:

AM	amniotic membrane
ANG	angiogenin
BM-MSCs	bone marrow MSCs
CD	cluster of differentiation
CM	conditioned medium
CSK	corneal stromal keratinocyte
CT	computed tomography
DAMPs	damage-associated molecular patterns
dHACM	amnion/chorion membrane allograft
dhAM	decellularized hAM
DoD	drop-on-demand
EAE	experimental autoimmune encephalomyelitis
ECM	extracellular matrix
EGF	epidermal growth factor
EV	extracellular vesicle
FGF	fibroblast growth factor
GO	graphene oxide
GRO	growth-related oncogene
GV	graft versus leukemia
GVHD	graft versus host disease
hADSCs	human adipose-derived stem cells
hAECs	hAM epithelial cells
hAM	human amniotic membrane
hAMSCs	hAM mesenchymal stromal cells
hAoECs	human aorta endothelial cells
hCECs	human corneal epithelial cells
hCSCs	corneal stem cells
HGF	hepatocyte growth factor
HLA	human leukocyte antigen
HSCT	post-hematopoietic stem cell transplantation
HUVECs	human umbilical vein endothelial cells
IDO	indoleamine 2, 3-dioxygenase
IUA	intrauterine adhesion
IFN-γ	interferon γ
IL	interleukin
imMKCL	immortalized megakaryocyte progenitor cell line
iPSC-ECs	induced pluripotent stem cell-derived endothelial cells
KGF	keratinocyte growth factor
LncRNAs	long non-coding RNAs
lsEVs	large-size EVs
MCP1	monocyte chemoattractant protein1
M-CSF	macrophage colony-stimulating factor
miRNA	micro-RNA
MKCLs	human iPSC-derived megakaryocytes
MPO	neutrophil myeloperoxidase
MRI	magnetic resonance imaging
MSCs	mesenchymal stromal/stem cells
NET	neutrophil extracellular trap
NGF	nerve growth factor
NK	natural killer
PAVICs	porcine aortic valve interstitial cells
PBMCs	peripheral blood mononuclear cells
PCL	polycaprolactone
PDGF	platelet-derived growth factor
PEDF	pigment epithelium-derived growth factor
PEGDA	poly-ethylene glycol-diacrylate
PGE	prostalglandin
ssEVs	small-size EVs
TGF-β	transforming growth factor β
Treg	regulatory T cell
TIMPs	tissue inhibitors of metalloproteinases
TNF-α	tumor necrosis factor α
VEGF	vascular endothelial factor
WJ-MSCs	Wharton’s jelly-derived MSCs
